# Supplementary material for: Editing the RR-TZF Gene Subfamily in Rice Uncovers Potential Risks of CRISPR/Cas9 for Targeted Genetic Modification
Source: Int J Mol Sci. 2025 Feb 5;26(3):1354. doi: 10.3390/ijms26031354 (PMC11818430; doi:10.3390/ijms26031354)
Supplement: Supplementary file 1 [file ijms-26-01354-s001.zip › ijms-3374914-supplementary.pdf]

Table S1. Target sites of rice *RR-TZF* genes for CRISPR/Cas9-mediated gene editing.

Gene	Locus	Target Name	Position	Direction	Target Sequence (5'−3')[1]
OsTZF1	LOC_Os05g10670	KTZF1	41-63bp	-	GAGGCCCCCTCCGCTAACCGCGG
OsTZF2	LOC_Os01g09620	KTZF2	51-73bp	-	GTACATCGACTCCGCTCACGGGG
OsTZF3	LOC_Os03g49170	KTZF3	399-421bp	-	AGCCACCAGACGCCGCACAGTGG
OsTZF4	LOC_Os12g33090	KTZF4	181-203bp	-	GCAGCCCACCGAGCCGTACACGG
OsTZF5	LOC_Os05g03760	KTZF5	133-155bp	+	GAGGAGGAGAAGGTGTCTCTTGG
OsTZF6	LOC_Os07g38090	KTZF6	463-485bp	-	TCGAGATCGCCAAGGGCATCTGG
OsTZF7	LOC_Os05g45020	KTZF7	48-70bp	+	CTCTTGGGCTGACCCCGCTGCGG
OsTZF8	LOC_Os01g53650	KTZF8a	45-67bp	+	GGAGTTTCTAGCCGCGCTGCAGG
		KTZF8b	270-292bp	-	GGGCGGCGACGCGGAAGTCCGGG
OsTZF9	LOC_Os07g47240	KTZF9a	120-144bp	+	TCTCTGGGCATCCATGTCGGAGG
		KTZF9b	332-354bp	-	GTAGCGGCAACGGAGGCCGTGGG
		KTZF9c	356-378bp	+	CCCATGGTGTCTTCGAGCTATGG

The protospacer adjacent motif (PAM) sequences are shown with underlines.

Table S2. Editing types of targeted mutations in T_0 transgenic plants.

Target	No. of edited sites	Insertion	Deletion	Substitution	Complex
KTZF1	35	20（57.1%）	11（31.4%）	0	4（11.4%）
KTZF2	38	11（28.9%）	25（65.8%）	0	2（5.3%）
KTZF4	56	25（44.6%）	30（53.6%）	0	1（1.8%）
KTZF5	59	51（86.4%）	8（13.6%）	0	0
KTZF6	56	16（28.6%）	32（57.1%）	1（1.8%）	7（12.5%）
KTZF7	26	13（50.0%）	10（38.5%）	0	3（11.5%）
KTZF8a	39	29（74.4%）	9（23.1%）	0	1 (2.6%)
KTZF8b	64	13（20.3%）	43（67.2%）	0	8 (12.5%)
KTZF9b	14	2（14.3%）	9（64.3%）	0	3 (21.4%)
KTZF9c	29	2（6.9%）	25（86.2%）	0	2 (6.9%)
Total	416	182（43.8%）	202（48.6%）	1（0.2%）	31 (7.4%)

Table S3. Primers for target site sequencing.

Primer name	Primer sequence (5'−3')	PCR product size (bp)
KTZF1F2	TTTTCGCCTTGGGCTGTA	696
KTZF1R2	GCCGCACCTTGAACTCGTA	
KTZF2F	TCGCCTTACACATCGCACCA	554
KTZF2R	GAGCCAACACTCAAACACGCC	
KTZF3F2	ATGGCGAAAACCCTCACC	436
KTZF3R2	GGGCAATCTTGGCGTCA	
KTZF4F2	TCCCTTTCCCCATCTCCTCC	775
KTZF4R2	CACATGTCCCCTCTCCTGCA	
KTZF5F2	AACCCCCATTCCTCCATTACTC	868
KTZF5R2	GGTGCTGAACAGTCCGCTCTT	
KTZF6F2	ATGGGGTCGGGCTGTGGTAT	665
KTZF6R2	AGTGTATGGGTGCTTGCGGG	
KTZF7F1	TTTCTTCGGTGGTGTTGGGA	702
KTZF7R1	TGCGAACATCAACTCTTCCATC	
TZF8F	CTCATCTAATCCAACGCCACC	715
TZF8R	CAAGTTTCACATCAACAGGTCA	
KTZF9F1	CGGAGGATTTCTGGATACACG	372
KTZF9R1	TGAGGATGGCTGACGCAAT	

Table S4. Primers for off-target analysis.

Target site	Primer name and sequence (5'−3')	PCR product size (bp)
KTZF1	1OTF1: CTCAATCGGAACCGCACA	570
	1OTR1: CCCAAAAATGACAACAGAAGGA	
KTZF1	1OTF2: TTCTCCCCTTCCTTCCCCTTTT	405
	1OTR2: CACAACCCATCTCCACCATCCC	
KTZF1	1OTF3: ACGCACCCACACCACACAA	393
	1OTR3: AAGGAGCCGCCGTGGATGTA	
KTZF1	1OTF4: AGGGGAAAGGGAAAGAAGG	774
	1OTR4: GCTCGTGTTTCGCATTGG	
KTZF2	2OTF1: TCTCCAGCAATCAGATGTGTC	418
	2OTR1: ACCCCATCACCTCCTTATTT	
KTZF2	2OTF2: AAAATGACCACATCAGCCT	441
	2OTR2: ACACGACGACACAAAGCAG	
KTZF2	2OTF3: TGCGTCTGCGGCTGATTTC	594
	2OTR3: TGGGTGTAGGGCTTTCGGTC	
KTZF4	4OTF1: CTCGTCGGCTTCCACACCTT	592
	4OTR1: GGCACACCGTGTAGATGTAGGG	
KTZF4	4OTF2: AAACCGTTGATACGATGAGAGCA	594
	4OTR2: TTCTACTTCCACCGCCCCTC	
KTZF4	4OTF3: GCCAAGACCAAACTGCCAAGG	596
	4OTR3: CCTCCTTCCCAATGTCGCACT	
KTZF4	4OTF4: CGCCTCGTGAAGGAGAACA	509

	4OTR4: TTGACGAAGTTGCCGAGGG	
KTZF5	5OTF1: AAACCCTAACCCAGCATCAC	484
	5OTR1: TGAGCCTAAGCACTCTTCGTT	
KTZF5	5OTF2: TCCACCCTTGCTCGTTTTG	460
	5OTR2: GAAGGATTGGGGAGGCATTT	
KTZF5	5OTF3: ACCTCACCGAAGTCATCCC	557
	5OTR3: CATTTGTGTTGTTCTCACTCAG	
KTZF5	5OTF4: AGGCGGGGAAGGTAACAGGT	590
	5OTR4: GGATGAGACGGCACGACAAC	
KTZF5	5OTF5: GGGTTAGGGTTTGGCGTGA	538
	5OTR5: GGAGGGGAAAGACAGGGTGATA	
KTZF6	6OTF1: CCTCCCACGGCATCATCAA	481
	6OTR1: TCGCAAGTCCCAACCATCC	
KTZF6	6OTF2: TCGGTGAGGACATCGCCAA	408
	6OTR2: GGGTAACAAGAAAGACGCATT	
KTZF6	6OTF3: AGGTCCTATTTGGCTTGGC	506
	6OTR3: TGGTGATGCTCTAAGGCTGTC	
KTZF7	7OTF1: CCAAAACTTTACACCCCCA	543
	7OTR1: CAATCAACCATTCTGCCAT	
KTZF7	7OTF2: GGCAGCAATCCCCGAAAA	514
	7OTR2: GGCGGAAGAACAGGAACGAC	
KTZF7	7OTF3: GATGACGATGTCGCCCCACT	542
	7OTR3: CGTGTCTCACTCCTGCACACAC	
KTZF7	7OTF4: TCCTCGTCGTAGTCGCTGTTGC	735
	7OTR4: CCCCTTTCCCTTCCACTTCTCC	
KTZF7	7OTF5: CATTTCATTTGGCTTCTACCTC	792
	7OTR5: AATCCGTGGACCCTCATCT	
KTZF8a	8aOTF1: AGGGACAACAAATCGTGGCG	538
	8aOTR1: TGAAGGACGGCAACCTGGAG	
KTZF8a	8aOTF2: TTATCACCACCAATAGTTCAGG	504
	8aOTR2: GTTCGTCATCAAACTCCGC	
KTZF8a	8aOTF3: TGGAACGGTTTCACTATGGACG	388
	8aOTR3: GCGAGGACGATGGTGTTGAAT	
KTZF8b	8bOTF1: AGCCCTCTCGGGATGACAA 3'	549
	8bOTR1: GCTCGTCTCCTCACTGCTACTG	
KTZF8b	8bOTF2: GTGAAGGAAAAGGGTTGGAGA	502
	8bOTR2: TGTCTGGCAGGAGGTGGTAG	
KTZF8b	8bOTF3: TCAGTTGCTTAAAATCTCCCCA	643
	8bOTR3: GTGACGAAGCCGAAGTAGACG	
KTZF8b	8bOTF4: AAGGTTCAGATTAGCCCCG	635
	8bOTR4: CCACCGTGCCCTTTACAT	
KTZF8b	8bOTF5: GCACGGACCACCTTGGAGTA	430
	8bOTR5: CTGGAGTGGTGATCGTCGTG	
KTZF9b	9bOTF1: GCGAAAGCAATAAACCATCC	357

	9bOTR1: AAAGGCGACGAGCAGAAG	
KTZF9b	9bOTF2: TAGGGAAAATGTCGCTCTTGCC	638
	9bOTR2: CAAATGCCCTGGAACCATCAAG	
KTZF9b	9bOTF3: TTTGGAGATTGTTTTGGCTGACG	458
	9bOTR3: AGGAGGACGGCGTGCTTCAT	
KTZF9b	9bOTF4: AGTTTTGGGACGGCGAGA	592
	9bOTR4: CTTGCTTCGTCATCGGCT	
KTZF9b	9bOTF5: TCCGCAAGAACAGAACGAA	885
	9bOTR5: AAAACCGACGCCTATTGAAA	
KTZF9c	9cOTF1: CCCCCACAAACCAATGAG	575
	9cOTR1: AGTTTGCCGAATCCAGCG	
KTZF9c	9cOTF2: AGCACGAAGTTGCCACTATCC	498
	9cOTR2: GCCCATCTTAGTAGGAGCCG	
KTZF9c	9cOTF3: CTCGTGTAGTGGACATAGCA	571
	9cOTR3: TTACCCGTCATACCACACC	
